# Safety and Pharmacokinetics of the Substance of the Anti-Smallpox Drug NIOCH-14 after Oral Administration to Laboratory Animals

**DOI:** 10.3390/v15010205

**Published:** 2023-01-11

**Authors:** Larisa N. Shishkina, Oleg Yu. Mazurkov, Nikolai I. Bormotov, Maksim O. Skarnovich, Olga A. Serova, Natalia A. Mazurkova, Maria A. Skarnovich, Alexander A. Chernonosov, Boris A. Selivanov, Alexey Ya. Tikhonov, Svetlana G. Gamaley, Galina G. Shimina, Galina M. Sysoyeva, Oleg S. Taranov, Elena D. Danilenko, Alexander P. Agafonov, Rinat A. Maksyutov

**Affiliations:** 1 Federal Budgetary Research Institution—State Research Center of Virology and Biotechnology VECTOR, Federal Service for Surveillance on Consumer Rights Protection and Human Well-Being, 630559 Koltsovo, Russia; 2 The Institute of Chemical Biology and Fundamental Medicine, Siberian Branch of the Russian Academy of Sciences, 630090 Novosibirsk, Russia; 3 N.N. Vorozhtsov Novosibirsk Institute of Organic Chemistry of the Siberian Branch of Russian Academy of Sciences, 630090 Novosibirsk, Russia

**Keywords:** orthopoxviruses, anti-smallpox drugs, NIOCH-14, safety, pharmacokinetics

## Abstract

Background: Since most of the modern human population has no anti-smallpox immunity, it is extremely important to develop and implement effective drugs for the treatment of smallpox and other orthopoxvirus infections. The objective of this study is to determine the main characteristics of the chemical substance NIOCH-14 and its safety and bioavailability in the body of laboratory animals. Methods: The safety of NIOCH-14 upon single- or multiple-dose intragastric administration was assessed according to its effect on the main hematological and pathomorphological parameters of laboratory mice and rats. In order to evaluate the pharmacokinetic parameters of NIOCH-14 administered orally, a concentration of ST-246, the active metabolite of NIOCH-14, in mouse blood and organs was determined by tandem mass spectrometry and liquid chromatography. Results: The intragastric administration of NIOCH-14 at a dose of 5 g/kg body weight caused neither death nor signs of intoxication in mice. The intragastric administration of NIOCH-14 to mice and rats at doses of 50 and 150 µg/g body weight either as a single dose or once daily during 30 days did not cause animal death or critical changes in hematological parameters and the microstructure of internal organs. The tissue availability of NIOCH-14 administered orally to the mice at a dose of 50 µg/g body weight, which was calculated according to concentrations of its active metabolite ST-246 for the lungs, liver, kidney, brain, and spleen, was 100, 69.6, 63.3, 26.8 and 20.3%, respectively. The absolute bioavailability of the NIOCH-14 administered orally to mice at a dose of 50 µg/g body weight was 22.8%. Conclusion: Along with the previously determined efficacy against orthopoxviruses, including the smallpox virus, the substance NIOCH-14 was shown to be safe and bioavailable in laboratory animal experiments.

## 1. Introduction

The past few decades have witnessed an increased frequency and severity of epidemic outbreaks of orthopoxvirus infections such as those caused by the cowpox and monkeypox viruses [1,2,3,4,5,6,7]. The loss of herd immunity against smallpox and other orthopoxvirus infections in people (due of the cessation of anti-smallpox vaccination more than 40 years ago) has laid the groundwork for the spreading of zoonotic orthopoxviruses, possibly promoting the natural selection of viral variants that are highly pathogenic and epidemiologically threatening. Therefore, recent efforts of the World Health Organization (WHO) have focused on designing chemotherapeuticals that would be effective against the variola virus and other pathogenic orthopoxviruses [8]. Since there was a monkeypox outbreak among humans in more than 100 countries from the six WHO regions in 2022, the relevance of developing and implementing effective chemotherapeuticals against orthopoxvirus infections has significantly increased [9].

Tecovirimat (TPOXX), based on the chemical compound ST-246, is a drug for the treatment of orthopoxvirus infections in humans that has been FDA registered and approved for application in the USA [10]. The anti-orthopoxviral chemical compound ST-246 was obtained and described in 2005 in the USA [11]. It inhibits the final stage of the assembly of enveloped virions and prevents virus exit from the infected cell [12]. ST-246 has exhibited low toxicity and high antiviral efficacy for mice infected with the ectromelia virus (ECTV); rabbits with the rabbitpox virus (RPXV); prairie dogs with the monkeypox virus (MPXV); and monkeys with the variola virus (VARV) and MPXV [13,14]. Experiments using different animals (mice, rabbits, and nonhuman cynomolgus monkeys) have also proved that ST-246 is safe [15]. Furthermore, the absolute oral bioavailability (Fabs) of ST-246 administered to cynomolgus monkeys at doses ranging from 3.0 to 30 µg/g, which had previously been used in efficacy studies, was evaluated. In general, ST-246 administered to monkeys exhibited good bioavailability ranging from 31 to 77% [15]. It is also known from the research literature that the Fabs of ST-246 administered to different mouse strains ranged from 4 to 72% [11,15].

An analog of ST-246 (the novel chemical compound) known as NIOCH-14 (having patent certification) was synthesized at the Novosibirsk Institute of Organic Chemistry, Siberian Branch of the Russian Academy of Sciences (NIOCH SB RAS) in 2009 in collaboration with the State Research Center of Virology and Biotechnology VECTOR of the Russian Federal Service for Surveillance on Consumer Rights Protection and Human Wellbeing [16]. Previously, we reported that this compound exhibited high activity in the treatment of orthopoxvirus infections in various animal models. Thus, the experiments using outbred ICR mice intranasally infected with a 100% lethal dose of the ectromelia virus have revealed that a 50% effective dose of NIOCH-14 administered orally to mice was 3.59 µg/g body weight and did not significantly differ from the respective dose of the ST-246 reference drug (5.08 µg/g). The “therapeutic window” (the time to initiate the daily administration of NIOCH-14 to ensure the effective protection of mice from death) varied from 1 day pre-infection to 6 days post-infection of ectromelia virus for achieving 100 to 60% survival, respectively, which significantly differed from that in the control groups. The administration of NIOCH-14 reduced virus production in the lung, nasal mucosa, brain, trachea, liver, spleen, kidney, and pancreas and also alleviated the microscopic pathological changes in the lungs of the animals 6 days post-infection [17,18]. The oral administration of NIOCH-14 to ICR outbred mice and bobak marmots infected with the monkeypox virus strain V79-1-005 significantly reduced virus production in the lungs and reduced the number of infected mice 7 days post-infection; no signs of disease in bobak marmots were observed compared to the control group [18]. The experiments demonstrated that the number of infected mice and the virus production in the lungs were significantly reduced after the oral administration of NIOCH-14 to immunocompetent ICR outbred mice and mice with SCID immunodeficiency infected with the Ind-3a variola virus strain 3- and 4-days post-infection, respectively [18]. The resulting findings suggest a great potential for the use of the substance NIOCH-14 as a platform to develop an anti-smallpox therapeutical to be produced in Russia [16,18,19].

A necessary step in the development of an effective Russian anti-smallpox drug based on the chemical compound (substance) NIOCH-14 is to study safety and bioavailability as well as such characteristics as the concentration and distribution of the substance administered to laboratory animals across their body tissues. Therefore, the aim of this study was to assess the safety and bioavailability of the chemically synthesized substance NIOCH-14 in animal experiments.

## 2. Materials and Methods

### 2.1. Samples

The chemical compound (substance) NIOCH-14 (7-({[(4-trifluoromethyl)phenyl]formohydrazido}carbonyl)tricyclo[3.2.2.0^2,4^]non-8-en-6-carboxylic acid), synthesized at the Vorozhtsov Novosibirsk Institute of Organic Chemistry, SB RAS, according to the procedure elaborated earlier and described in ref. [16,19], was used in this study. In terms of its chemical structure, the compound NIOCH-14 is the closest analog (or to be more specific, the precursor during synthesis) of ST-246 (N-{3,5-dioxo-4-azatetracyclo[5.3.2.0^2,6^.0^8,10^]dodec-11-en-4-yl}-4-(trifluoromethyl)benzamide), has patent certification [20], and exhibits a high anti-orthopoxvirus activity compared to that of ST-246 both in vitro and in vivo [21]. The substance NIOCH-14 is formed via the reaction between annelated succinic anhydride and hydrazide of 4-trifluoromethyl benzoic acid upon cooling [16]. The purity of the substance was no less than 94% and was determined by acid–base titration in non-aqueous media. A pharmacokinetic study of NIOCH-14 showed that it is insoluble in water and solvents commonly used in mass spectrometry (acetonitrile or alcohols); meanwhile, NIOCH-14 undergoes cyclization over time and the content of ST-246 increases in dimethyl sulfoxide (DMSO). In this solvent, as well as in blood serum, organ homogenates, and the animal organism, NIOCH-14 is converted to its active metabolite ST-246 and the secondary metabolite K (4-trifluoromethyl benzoic acid). The 2-hydroxy-N-{3,5-dioxo-4-azatetracyclo[5.3.2.0^2,6^.0^8,10^]dodec-11-en-4-yl}-5-methylbenzamide was used as an internal standard (N-98, IS).

Metabolite K is the metabolic product of ST-246 and NIOCH-14, thus being their secondary (inactive) metabolite. The concentration and pharmacokinetic parameters of the secondary metabolite K in mouse blood and organs are not reported in this study as they are non-informative for the assessment of the tissue and absolute bioavailability of NIOCH-14.

The chemical compound ST-246—having known anti-smallpox activity and having been synthesized for research purposes at the NIOCH SB RAS according to the procedure described in ref. [22]—was used as the reference drug and active metabolite of NIOCH-14. ST-246 is formed via the reaction between annulated succinic anhydride and hydrazide of 4-trifluoromethyl benzoic acid upon heating [22].

All chemical compounds were synthesized at the NIOCH SB RAS and were provided to us together with the certificates containing the information about their chemical names and main physicochemical characteristics (molecular weight, the empirical formula, the chemical structure and formula, the NMR spectra, etc.). The chemical names of the compounds are given under the nomenclature of the International Union of Pure and Applied Chemistry (IUPAC).

### 2.2. Animals

Thirty-six male and female ICR outbred mice (weight, 18–20 g) and thirty Wistar rats (males weighing 190–230 g and females weighing 160–200 g) were used for studying the acute toxicity of the substance NIOCH-14. Eighty male and female Wistar rats (weight, 160–200 g) were used to study the subchronic toxicity of NIOCH-14. A total of 280 male and female ICR outbred mice (weight, 12–14 g and 18–20 g, respectively) were used to study the pharmacokinetics of NIOCH-14. All the animals were procured from the laboratory animal nursery of the State Research Center of Virology and Biotechnology VECTOR of the Russian Federal Service for Surveillance on Consumer Rights Protection and Human Wellbeing. The animals were kept under natural light conditions, fed a standard diet, and had ad libitum access to water. All the manipulations with the animals, including anesthesia and euthanasia, were conducted in compliance with the current sanitary regulations and norms of SanR&N 3.3686-21 “Sanitary and epidemiological requirements for the prevention of infectious diseases” [23], “Guidelines on laboratory animal care and use” [24], and the Protocol of studies using laboratory animals approved by the Bioethic Commission of the State Research Center of Virology and Biotechnology VECTOR of the Russian Federal Service for Surveillance on Consumer Rights Protection and Human Wellbeing, No. IMBT/5-07.12 dated 13 July 2012.

### 2.3. Identifying the Lethal Doses of the Agent

According to the available literature data, ST-246 did not cause death in animals when administered orally at a dose of 2 g/kg body weight [25]. These data implied that NIOCH-14 can also be classified as a slightly or moderately hazardous compound. Therefore, we used a dose of 5 g/kg body weight, which is the boundary dose for these two classes of compounds, to determine the level of hazard of the agent according to the classification specified in the State Standard GOST 12.1.007-76 “The Occupational Safety Standards System. Noxious Substances. Classification and General Safety Requirements”. For this purpose, a NIOCH-14 suspension in an aqueous solution of methylcellulose (0.75%) and Tween 80 (1%), the content of the basic substance being 200 mg/mL, was administered intragastrically to six male ICR outbred mice portionwise for 1 h; the total volume was 0.5 mL, which corresponds to the dose of 5 g/kg (the maximum possible dose that can be administered with allowance for the maximum possible concentration of the drug and maximum possible volume that can be administered). The follow-up period, during which the animals’ death was documented, lasted 7 days. Euthanasia of the surviving animals and macroscopic pathomorphological examination of their internal organs were performed.

### 2.4. Evaluating Safety of NIOCH-14 upon Single-Dose Administration to Mice

The drug was administered intragastrically to ICR outbred mice in the study group as a single dose (150 µg/g body weight; this dose was threefold higher than the effective dose determined using the experimentally induced model of orthopoxvirus infection) [18] according to guidelines for conducting preclinical studies of drugs [26]. For technical reasons, this dose was the maximum possible dose for single-dose administration of the NIOCH-14 preparation as a homogeneous suspension using a gastric tube. The suspension of NIOCH-14 (see Section 2.3), the content of the basic substance being 15 mg/mL, was administered to the mice (0.2 mL per 20 g body weight). The animals in control group 1 received a single dose of normal saline (an identical volume) intragastrically; animals in control group 2 received an aqueous solution of methylcellulose (0.75%) and Tween 80 (1%). Each study group consisted of five male and five female mice. A daily follow-up was carried out 14 days after the administration of the drug; the animals’ appearance, mobility, behavioral characteristics, as well as feed and water consumption were evaluated. The clinical examination of the animals was performed according to the parameters listed above. Mice were withdrawn from the experiment by performing instantaneous cervical dislocation 14 days after drug administration, and blood samples were collected for hematological analysis. If there are no changes in hematological parameters 14 days after a single dose, then their possible presence at an earlier date is not critical, since they disappear anyway. The leukocyte, erythrocyte, and platelet counts and the hemoglobin level and hematocrit were determined in the blood samples using the automated hematology analyzer “Hemolux 19” (MINDRAY, China). The erythrocyte sedimentation rate (ESR) and types of white blood cells were determined using the standard procedures [27].

### 2.5. Evaluating Safety of NIOCH-14 upon Single-Dose Administration to Rats

The substance NIOCH-14 was administered to Wistar rats intragastrically as a single dose (150 µg/g; the dose threefold higher than the effective dose for mice). For technical reasons, this dose was the maximum possible dose for the single-dose administration of the agent as a homogeneous suspension using a gastric tube. The NIOCH-14 suspension (see Section 2.3) was administered to rats (1 mL per 200 g body weight). The control animals received an equivalent volume of solution (see Section 2.3). Each study group consisted of five male and five female animals. A daily follow-up was carried out 14 days after the administration of the drug; the animals’ appearance, mobility, behavioral characteristics, as well as feed and water consumption were evaluated. Rats were withdrawn from the experiment by performing decapitation after isoflurane inhalation anesthesia 14 days after drug administration, and blood samples were collected for hematological analysis (see Section 2.4).

### 2.6. Evaluating Safety of the Substance NIOCH-14 upon Repeated Administration to Rats

NIOCH-14 was intragastrically administered to Wistar rats daily for 30 days with two doses. The first dose was 50 µg/g (the dose corresponding to the effective dose determined by studying specific activity in mice) [18]. Since in the acute toxicity experiments the agent administered intragastrically did not cause animal death, it was impossible to determine the LD_50_ and 1/5 LD_50_ levels; therefore, the threefold effective dose (150 µg/g) was used as the second subtoxic dose. For technical reasons, this dose was the maximum possible dose for single administration of the agent as a homogeneous suspension. The suspension of NIOCH-14 (see Section 2.3), the content of the basic substance being 30 mg/mL, was administered to the rats (1 mL per 200 g body weight). Control animals received an equivalent volume of solution (see Section 2.3). Each study group consisted of 10 male and 10 female animals. The experiment duration was selected in accordance with the “Guidelines for conducting preclinical studies of drugs” [27] as the duration recommended for studying the subchronic toxicity of drugs to be used in clinical practice for 14 days. After 1 and 30 days after the end of the course of drug administration, the rats were withdrawn from the experiment by decapitation after isoflurane inhalation anesthesia; blood samples were collected for hematological analysis, and organ samples were collected for histological analysis (see Section 2.4). Organ samples from five animals in each study group were collected for histological analysis. Samples of the brain and bone marrow, heart, liver, kidney, lung, spleen, thymus, adrenal gland, esophagus, stomach, large and small intestines, bladder, pancreas, and testis (or uterus and ovary) were harvested for pathomorphological study. For histological study, small pieces of organs were fixed in a solution containing 40% formalin solution and the Eagle MEM culture medium without L-glutamine. Histological specimens were prepared using the standard paraffin technique. Cross-sections up to 5 µm thick were stained using Mayer’s hematoxylin and eosin.

### 2.7. Oral or Intravenous Administration of NIOCH-14 or ST-246 to Mice to Assess Their Bioavailability

Male and female ICR outbred mice (weight, 12–14 g) were used in the experiments to assess the tissue availability of the substance NIOCH-14. The NIOCH-14 suspension was administered to the mice as a single dose (50 µg/g body weight) once p/o in 0.2 mL of methylcellulose solution with Tween 80 (see Section 2.3) using a pipette dispenser. Six mice in each group were euthanized by cervical dislocation 1, 6, 9, 12, 15, 18, 24, and 48 h after p/o administration of the drug; blood (to obtain serum), lung, spleen, kidney, and brain samples (for obtaining organ homogenates) were collected. The concentration of ST-246 was then determined in serum and organ samples.

Male and female outbred ICR mice (weight 18–20 g) were used for the experiments to assess the absolute bioavailability (F_abs_) of the drugs. Suspensions of the compounds NIOCH-14 (the substance) or ST-246 (the reference drug) were administered to mice orally (p/o) as a single dose of 50 µg/g body weight in 0.2 mL of a solution of methylcellulose and Tween 80 (see Section 2.3). Six mice in each group were euthanized by cervical dislocation 1, 3, 6, 9, 12, 15, 18, 24, and 48 h after p/o administration of the drug, and blood samples for obtaining serum were collected. The ST-246 concentration was then determined in mouse serum samples.

In order to assess the absolute bioavailability (F_abs_) of the drug, one needs to measure its pharmacokinetic parameters upon intravenous (i/v) administration. For this purpose, mice received a single i/v dose of the substance NIOCH-14 or the reference drug ST-246 at a dose of 2 µg/g in 0.1 mL of a solution containing 2% DMSO, 50% PEG-400, 20% methanol, and 1% Tween 80. The solution of the drug was injected into the tail vein of the mice, and no visible adverse reactions of the animals to the i/v administration were observed. Since NIOCH-14 and ST-246 are water-insoluble, the composition of the solvent used in this experiment was chosen in accordance with the guidelines for studying the bioavailability of ST-246 [11,25]. Six mice in each group were euthanized by cervical dislocation 0.25, 1, 3, 6, 9, 12, 15, 18, and 24 h after the i/v administration of the drugs, and blood samples for obtaining serum were collected. The concentration of ST-246 was then determined in serum samples.

### 2.8. Justification of Using a Concentration of the Active Metabolite ST-246 to Describe the Bioavailability of NIOCH-14

The substance NIOCH-14 is water-insoluble and poorly soluble in solvents commonly used for mass spectrometry (acetonitrile and alcohols). At concentrations required for plotting the calibration curves, NIOCH-14 is soluble in 10% DMSO in acetonitrile. In this solvent, NIOCH-14 is converted to its active metabolite ST-246. In the mass spectrum, the peaks with 393 *m*/*z* and 375 *m*/*z* correspond to NIOCH-14 and ST-246, respectively; the peak with 337 *m*/*z* corresponds to the control substance (the internal standard) N-98 (where *m*/*z* is the molecule mass-to-charge ratio). When NIOCH-14 is added to the blood serum or organ homogenates of mice, it is completely converted to the active metabolite ST-246 during the extraction and express test (the total time being no longer than 1 h), and the peak with 393 *m*/*z* corresponding to NIOCH-14 cannot be detected in the analyzed sample. Because of the conversion of NIOCH-14 to the primary active metabolite ST-246, its concentration recorded, depending on the time after administration of the substance, can characterize the pharmacokinetic parameters of NIOCH-14 in the mouse organism.

### 2.9. Sample Preparation and Extraction Procedure

Fifty-percent organ homogenates were obtained by adding distilled water in a 1:1 ratio to the organ weight. The homogenates were centrifuged for 15 min at 2000× *g* in Eppendorf MiniSpin centrifuge (Eppendorf AG, Hamburg, Germany), and the supernatants were collected in another tube. The extraction of ST-246 from the mouse serum and mouse organ homogenate was slightly different.

In the case of blood serum, sample preparation was carried out as follows:

1. To 80 µL of blood serum, 4 µL of internal standard solution (10 µg/mL) was added. The samples were incubated for 30 min on a Mixer 5432 shaker (Eppendorf, Germany).
2. Then, the 100 µL of methanol and 250 µL of acetonitrile were added in succession to precipitate proteins and extract the ST-246. Extraction was carried out by shaking samples on a Mixer 5432 (Eppendorf, Germany) for 60 min at room temperature.

After centrifugation at 10,000× *g* for 15 min, the 300 μL of supernatant was transferred into vials for subsequent LC/MS analysis.

In the case of the homogenates of mouse organs (kidneys, liver, and lungs), sample preparation was carried out as follows:

3. To 80 µL of homogenate, the 5 µL of internal standard solution (10 µg/mL) was added. The samples were incubated for 30 min on a Mixer 5432 shaker (Eppendorf, Germany).
4. Then, the 120 µL of methanol and 300 µL of acetonitrile were added in succession to precipitate proteins and extract the ST-246. Extraction was carried out by shaking samples on a Mixer 5432 (Eppendorf, Germany) for 60 min at room temperature.

After centrifugation at 10,000× *g* for 15 min, the 300 μL of the supernatant was transferred into vials for subsequent LC/MS analysis.

### 2.10. LC-MS Analysis in SRM and MRM Modes

The concentration of ST-246 in the blood serum and organ homogenates of the mice was determined by the LC-MS or LC-MS/MS methods. The mass-spectrometric analysis was carried out at the Core Facility of Mass Spectrometric Analysis at the Institute of Chemical Biology and Fundamental Medicine, the Siberian Branch of the Russian Academy of Sciences. The mass-spectrometric analysis was performed on a QQQ Agilent 6410 mass spectrometer (Agilent Technologies, Santa Clara, CA, USA) coupled to an Agilent 1200 HPLC system (Agilent Technologies, Santa Clara, CA, USA). The MassHunter v. 3.1 software (Agilent Technologies, Santa Clara, CA, USA) was employed for setting up the analysis and for data management.

The direct-inject method was used without chromatographic separation, and only the Zorbax Eclipse XBD-C18 guard column (4.6 × 12.5 mm, 5 μm) was applied to reduce contamination of the mass spectrometer. The mobile phase was composed of eluent A (water) and eluent B (acetonitrile). Analysis was performed at 200 μL/min in a 3.5 min run using the isocratic gradient—80% of buffer B.

The analysis parameters in both modes were set as follows: the volume of the aliquot, 50 μL; capillary voltage, 4 kV. Nitrogen was supplied as a spray and carrier gas under a pressure of 45 psi at a flow rate of 6 L/min at 300 °C. The dwell time was set to 200 ms, delta EMV to 200 V, and the Q1 and Q3 resolution to “unit”.

Mass-spectrometric analysis was performed in selected reaction monitoring (SRM) or multiple reaction monitoring (MRM) mode by electrospray ionization (ESI) in negative mode.

The measurements were carried out in the SRM mode for ions with *m*/*z* 375 (ST-246) and 337 (IS) when determining the pharmacokinetic parameters of the active metabolite ST-246 in the blood serum and organ homogenates of the mice that orally received NIOCH-14. The MRM mode was used for precursor ions with *m*/*z* 375.1 (ST-246) and 337.2 (IS) when determining the absolute bioavailability of the active metabolite ST-246 in blood serum upon intravenous and oral administration of NIOCH-14. The transitions were selected and used for quantitative confirmation of a compound: *m*/*z* 375.1 → 283.1 (ST-246, collision energy 20 eV, fragmentation voltage 135 V) and *m*/*z* 337.2 → 245.2 (IS, collision energy 20 eV, fragmentation voltage 135 V).

The linearity of the method was evaluated in the range of 50 ng/mL–40 µg/mL in SRM mode, and 20 ng/mL–40 µg/mL in MRM mode; the concentration of active metabolite ST-246 was determined with an accuracy of ±20% for the low limit of quantification and ±15% for other calibration standards.

### 2.11. Processing the Results and the Method for Calculating the Pharmacokinetic Parameters of the Active Metabolite ST-246

For data acquisition followed by calculation of the pharmacokinetic parameters of the substance NIOCH-14 using the add-in program in Microsoft Excel (PKSolver) [28], we plotted the time dependence of changes in concentration of the active metabolite ST-246. Origin 8.1 and Excel software were used for the statistical data analysis of the data and the plotting of the dependence curves.

The following significant pharmacokinetic parameters were eventually obtained:

1. Half-life of the drug T_1/2_ (h)—time during which drug concentration in blood (organ) decreases by 50%; this value is constant and independent of the dose or initial (maximum) drug concentration in blood (organ).
2. Time to reach the maximum concentration T_max_ (h)—time during which the maximum drug concentration in blood (organ) is reached.
3. The maximum concentration C_max_ (ng/mL)—the maximum drug concentration in blood (organ).
4. AUC (h × ng/mL)—area under the curve showing changes in drug concentration as a function of time (area under the curve “Concentration-Time”) from the instant of administration (0 h) to ∞ (AUC_0-inf_) or in the time interval from 0 h to time t that has passed since the administration of the compound (AUC_0-t_), in blood (organ).
5. Tissue availability (f_T_) for organs (%)—penetration of the compound into tissues calculated using the formula f_T_ = AUC_0-inf-T_:AUC_0-inf-S_, where AUC_0-inf-T_ is the area under the curve “Concentration-Time” from 0 to ∞ in the organ tissue; AUC_0-inf-S_ is the area under the curve “Concentration-Time” from 0 to ∞ in serum.
6. The absolute bioavailability (F_abs_) is the portion of drug that has reached systemic circulation, calculated using the formula F_abs_ = (AUC_0-t,p/o_ × D_i/v_):(AUC_0-t,i/v_ × D_p/o_), where AUC_0-t,p/o_ is the area under the curve “Concentration-Time” from 0 to t (time of complete disappearance) in serum upon oral (p/o) administration of the drug; AUC_0-t,i/v_ is the area under the curve “Concentration-Time” from 0 to t in serum upon intravenous (i/v) administration of the drug, D_p/o_ and D_i/v_ are the drug doses administered p/o and i/v, respectively.

### 2.12. Statistical Analysis

In the experiments to assess the effect of the substance NIOCH-14 on hematological parameters of laboratory animals, statistical analysis of the data was performed using the “Statgraphics, Vers 5.0” statistical software package (Statistical Graphics Corp., Warrenton, VA, USA). The group parameters of the pooled statistics (the arithmetic mean and mean error) were calculated. The normality of the parameter distribution was evaluated using the Shapiro–Wilk test. The data are presented as M ± m, where M is the mean value and m is the mean error. When studying the bioavailability of the substance NIOCH-14, the statistical analysis of the data and the plotting of the curves showing the time dependence of concentration of the active metabolite of NIOCH-14 (ST-246) were performed using Origin 8.1 and Excel software. The data are presented as M ± SD, where M is the mean value and SD is the standard deviation. The significance of intergroup differences in the normally distributed parameters were assessed using the Student’s *t*-test; otherwise, the Mann–Whitney U test was used. The critical significance level for testing the statistical hypotheses (*p*) was taken to be 0.05.

## 3. Results

### 3.1. Evaluation of Lethal Dose Values of the Substance NIOCH-14

This experiment revealed that a single-dose intragastric administration of the substance NIOCH-14 at a dose of 5 g/kg did not cause death in laboratory mice. The daily clinical examination of the animals (for 7 days) found no signs of intoxication, body weight loss, or reduction of body weight gain. The substance NIOCH-14 caused no macroscopic pathomorphological changes in the mouse organs 7 days after single-dose administration at a dose of 5 g/kg. These results allow one to classify it as a low-hazard compound for this administration route (in accordance with the State Standard GOST 12.1.007-76).

### 3.2. The Effect of the Substance NIOCH-14 on Hematological Parameters of Laboratory Animals

The study of the effect of the substance NIOCH-14 on the hematological parameters of the mice demonstrated that 14 days after a single-dose administration of the NIOCH-14 suspension at a dose of 150 µg/g, the white blood cell (WBC) count in the blood of the male mice was moderately reduced (by 23% vs. control group 1), while the WBC differential remained unchanged (Table 1). In the study group of female mice, the platelet count was moderately increased (by 28% vs. control group 1). There was no effect of the substance NIOCH-14 on red blood cell (RBC) count, hemoglobin level, hematocrit parameters, or erythrocyte sedimentation rate in the blood of the male and female mice (Table 1).

The study showed that 14 days after a single-dose administration of the substance NIOCH-14 at a dose of 150 µg/g body weight, there were no significant changes in hematological parameters in the male and female rats (Table 2). When studying the subchronic effect of the substance NIOCH-14 in the experiments in rats, the drug was administered at two doses (50 and 150 µg/g body weight) to rats once daily for 30 days (Table 3).

The findings demonstrated that the substance NIOCH-14 administered intragastrically at multiple doses (50 and 150 µg/g body weight) had no significant effect on the hematological parameters in male and female rats both 1 day (Table 3) and 30 days (data are not shown) after drug administration had been stopped.

### 3.3. The Effect of the Substance NIOCH-14 on the Pathomorphological Structure of Internal Organs of Laboratory Rats

**Figure 1:**
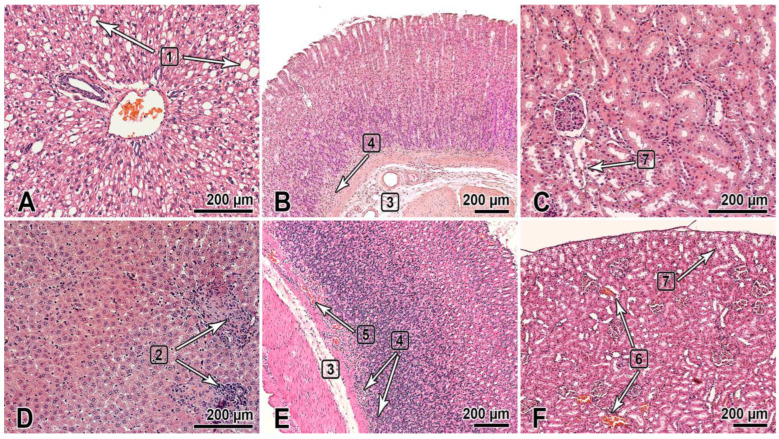
The organs of rats 1 day after the multiple-dose administration of the substance NIOCH-14 (150 µg/g body weight) once daily during 30 days was stopped. (**A**–**C**)—males; (**D**–**F**)—females. The bar is shown in the images. Hematoxylin and eosin staining. The liver (**A**,**D**): dystrophic changes in hepatocytes (steatosis) (1), foci of post-necrotic changes—fibrosis and lymphocytic infiltration (2). The gastric fundus mucosa (**B**,**E**): Gastric submucosa edema (3), mononuclear cell infiltration of the deep mucosa (4), and hyperemia (5). The renal cortex (**C**,**F**): hyperemia of renal cortical vessels (6) and vacuolar dystrophy with degeneration of the cytoplasm in the renal proximal tubular epithelium (7).

This section of the paper summarizes the results of the histological examination of the internal organs of the male and female rats that received the substance NIOCH-14 at doses of 50 and 150 µg/g body weight once daily for 30 days (Figure 1). The histological specimens of the male rats that received NIOCH-14 at a dose of 150 µg/g had alterations in the liver tissues such as hyperemia and dystrophic-necrotic changes in hepatocytes (parenchymal fatty dystrophy, small and medium drip) (2 of 5) (Figure 1A). They were detected in both groups receiving NIOCH-14 but were most clearly visible in the group of female rats that received NIOCH-14 at a dose of 150 µg/g (2 of 5) (Figure 1D).

Furthermore, two male rats in both groups that received NIOCH-14 at doses of 50 µg/g (1 of 5) and 150 µg/g (1 of 5) had changes in the mucosa of the gastric fundus, presenting as edema and the diffuse infiltration of the gastric submucosa by mononuclear cells (Figure 1B). Signs of gastric mucosal hyperemia and signs of mononuclear cell infiltration of the gastric submucosa, as well as moderate focal desquamation of the surface epithelium were found in female rats (2 of 5) (Figure 1E). This pathology was most severe in the study group of rats treated with NIOCH-14 at a dose of 150 µg/g (Figure 1B,E).

Pathological changes were also observed in the animals’ urinary system and presented as dystrophic-necrotic changes in the renal cortical epithelium in the male and female rats (Figure 1C,F). All these changes were observed in both study groups receiving NIOCH-14 at a dose of 50 µg/g (2 of 5 males, 1 of 5 females) and 150 µg/g (2 of 5 males, 2 of 5 females). Microcirculatory disorders, venous hyperemia, and vacuolar dystrophy with degeneration of the cytoplasm were observed in the renal parenchyma in both study groups, being more severe in rats treated with 150 µg/g NIOCH-14 (Figure 1C,F).

Therefore, the long-term administration of the substance NIOCH-14 over 30 days had an effect on the histological structure of the liver and the mucosa of the gastric fundus and renal cortex in rats. All the observed pathomorphological changes were more pronounced in the group of animals treated with 150 µg/g NIOCH-14.

The repeated intragastric administration of NIOCH-14 at any studied dose (50 or 150 µg/g body weight) had no effect on the histological structure of the other organs of the rats (the lungs, heart, pancreas, spleen, thymus, brain and spinal cord, small and large intestine, bladder, or adrenal glands). The microscopic appearances of these organs were identical in all the study and control groups.

### 3.4. Determining Tissue Availability (f_T_) of the Substance NIOCH-14

The concentration of active metabolite ST-246 in the serum and organs of the outbred ICR mice (weight, 12–14 g) after oral administration of the substance NIOCH-14 in three different modes was determined at the first stage.

**Figure 2:**
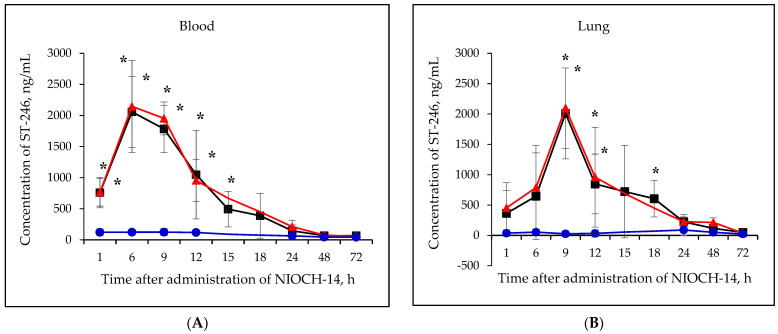

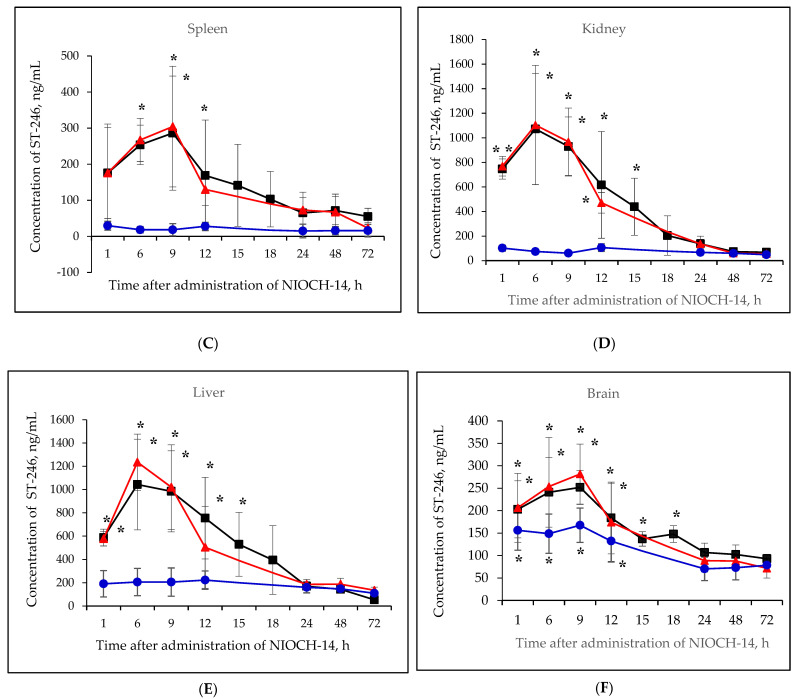
Changes in concentration of the active metabolite of NIOCH-14 (ST-246) in the mouse serum (**A**), lungs (**B**), spleen (**C**), kidneys (**D**), liver (**E**), and brain (**F**) after oral administration of the substance NIOCH-14. Single-dose administration of NIOCH-14 (50 µg/g body weight) (■). Administration of NIOCH-14 at a dose of 50 µg/g during 10 days (▲). Administration of NIOCH-14 at a dose of 5 µg/g during 10 days (●). Values in each point are presented as M ± SD. *—the difference from the threshold level (50 ng/mL) according to the Student’s *t*-test at *p* ≤ 0.05.

Figure 2A–F shows the pharmacokinetic curves of ST-246 after the single-dose oral administration of the substance NIOCH-14 (50 µg/g body weight) to mice, as well as after the oral administration of NIOCH-14 at doses of 50 and 5 µg/g body weight over 10 days. Figure 2A–F demonstrates that after administration of the substance NIOCH-14 at a dose of 5 µg/g body weight to mice over 10 days, concentration of the active metabolite ST-246 in all the analyzed animal organs, except for the brain, did not differ from the threshold level (50 ng/mL) at all time points after the administration had been stopped. In brain tissue (Figure 2F), the concentration of the active metabolite ST-246 was comparable to that observed 12 h after single-dose injection of the substance NIOCH-14 (50 µg/g). Furthermore, administration of the substance NIOCH-14 at a dose of 50 µg/g over 10 days compared to single-dose administration increased the concentration of active metabolite ST-246 in the serum and organs neither after 1, 6, and 9 h nor after 12 h and longer after the NIOCH-14 administration had been stopped (Figure 2A–F). Concentrations of the active metabolite ST-246 in serum and organs after administration of a single dose and ten doses of the substance NIOCH-14 (50 µg/g body weight) differed significantly from the threshold level for the time points indicated in the figure (Figure 2A–F).

The pharmacokinetic parameters of ST-246 in mouse serum and tissue organs after single-dose oral administration of the substance NIOCH-14 (50 µg/g body weight) were determined in this study. Table 4 summarizes the total calculated main pharmacokinetic parameters (T_1/2_, T_max_, C_max_, AUC_0-t_, AUC_0-inf_, and f_T_) of the active metabolite ST-246 in mouse serum and organs after single-dose oral administration of the substance NIOCH-14 in mice (50 µg/g body weight).

Table 4 shows the main pharmacokinetic parameters of ST-246 in mouse serum and organs after oral administration of the substance NIOCH-14 in mice in a single dose of 50 µg/g body weight. Therefore, it was revealed by studying the pharmacokinetic parameters of the substance NIOCH-14 administered orally in mouse experiments that this substance exhibits a tissue availability for the main organs (the lungs, heart, spleen, brain, and kidneys), which are the target organs for the ectromelia virus as shown previously [18].

### 3.5. Determining the Absolute Bioavailability (F_abs_) of the Substance NIOCH-14

Next, comparative studies of concentrations of the active metabolite ST-246 in mouse serum after single-dose oral administration of the substance NIOCH-14 (50 µg/g body weight) and its intravenous administration (2 µg/g body weight), as well as concentrations of the reference drug ST-246 for the same doses and routes of administration were conducted at the second stage.

**Figure 3:**
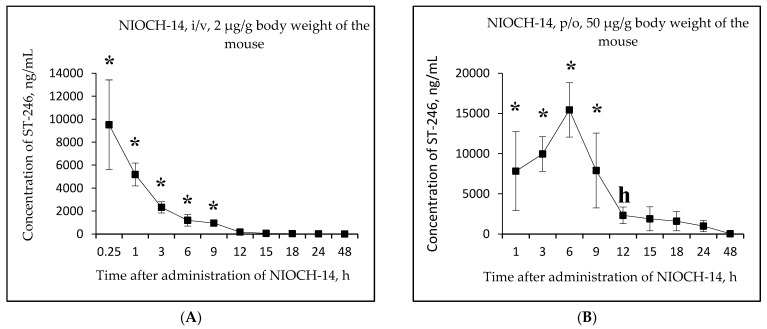

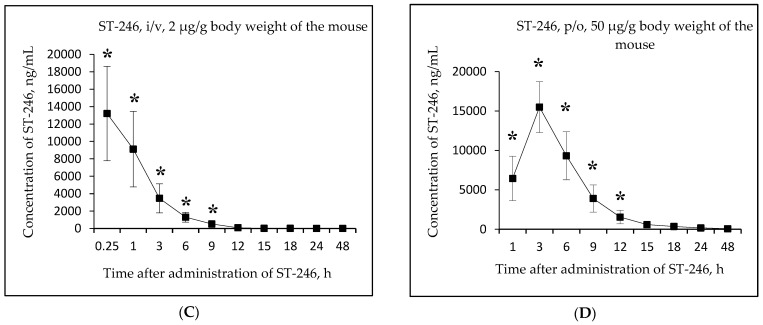
Changes in concentration of the active metabolite ST-246 in mouse serum as a function of time after single-dose intravenous (i/v) administration of the substance NIOCH-14 (2 µg/g body weight) (**A**) and single-dose oral (p/o) administration of the substance NIOCH-14 (50 µg/g body weight) (**B**). Changes in concentration of the reference drug ST-246 in mouse serum as a function of time after its single-dose i/v injection (2 µg/g body weight) (**C**) and single-dose p/o administration (50 µg/g body weight) (**D**). The values at each point are presented as M ± SD. *—the difference from the threshold level (20 ng/mL) according to the Student’s *t*-test at *p* ≤ 0.05.

Figure 3A,B shows the changes in the concentration of ST-246 in mouse serum as a function of time after single-dose intravenous injection of the substance NIOCH-14 (2 µg/g body weight) (Figure 3A) and after single-dose oral administration of the substance NIOCH-14 (50 µg/g body weight) (Figure 3B). Figure 3C,D shows the pharmacokinetic curves of the reference drug ST-246 in the mouse serum after single-dose intravenous injection of this substance (2 µg/g body weight) (Figure 3C) and after its single-dose oral administration (50 µg/g body weight) (Figure 3D).

Figure 3A,B shows that the maximum concentration of ST-246 in mouse serum was observed 15 min after intravenous and 6 h after oral administration of the substance NIOCH-14 (9.52 and 15.44 µg/mL, respectively). After intravenous administration of the reference drug ST-246, its maximum concentration in serum (13.20 µg/mL) was also observed after 15 min, whereas after oral administration of the reference drug ST-246, its maximum concentration (15.50 µg/mL) was reached after 3 h (Figure 3C,D).

Table 5 lists the calculated final pharmacokinetic parameters (T_1/2_, T_max_, C_max_, AUC_0-t_, AUC_0-inf_, and F_abs_) of the active metabolite ST-246 in mouse serum after single-dose intravenous and oral administration of the substance NIOCH-14 at doses of 2 and 50 µg/g body weight, respectively, as well as the reference drug ST-246 at the same doses and routes of administration. It was shown that the F_abs_ of the substance NIOCH-14 was 22.8% upon its oral administration at a dose of 2 µg/g body weight (Table 5). Furthermore, the pharmacokinetic parameters of the reference drug ST-246 in mouse serum were used to calculate its F_abs_, which was 12.1% upon oral administration at a dose of 50 µg/g body weight with respect to intravenous administration at a dose of 2 µg/g body weight (Table 5). Hence, the absolute bioavailability of the substance NIOCH-14 was found to be not lower than that of the reference drug ST-246.

## 4. Discussion

It should be noted that there are two drugs approved for the treatment of the diseases caused by orthopoxviruses; they are Tecovirimat (TPOXX, ST-246) and Brincidofovir [10,29]. The chemical compound NIOCH-14, being an analog of ST-246 but having patent certification, was synthesized at NIOCH SB RAS [16,19]. This paper summarizes the results obtained in preclinical studies of the chemically synthesized substance of the anti-smallpox drug NIOCH-14.

The chemical compound ST-246 exhibits antiviral activity against orthopoxviruses [11,25,30,31,32,33,34,35,36,37]. We have previously studied the specific activity of NIOCH-14 (substance) at different doses when infecting ICR outbred mice with the ectromelia virus (ECTV) [17,18]. Our previous data suggest that NIOCH-14, like ST-246, inhibits the systemic virus propagation and protects mice against lethal ECTV infection [17,18]. The plausible reason for that is as follows: because NIOCH-14 is a precursor of ST-246 during synthesis [16,19,20] and is converted to it in the body, it may have a similar mechanism of action; namely, it can inhibit the viral p37 protein, thus preventing replication of the ectromelia virus in the target organs and disease development [11].

In this study, we evaluated the safety of the substance of the candidate anti-smallpox drug NIOCH-14 in experiments using two types of laboratory animals: outbred ICR mice and Wistar rats. The experiment aiming to identify the lethal doses found that single-dose intragastric administration of the substance NIOCH-14 at a dose of 5 g/kg neither caused death in laboratory mice nor did it lead to weight loss, increase the rate of body weight gain, or induce any intoxication signs. Therefore, the substance NIOCH-14 can be classified as a low-hazard compound for this administration route. Because the parameters of lethal doses for intragastric administration could not be determined, an extended experiment was conducted to evaluate the safety of the substance NIOCH-14 at a dose of 150 µg/g, which exceeded the effective (therapeutic) dose in mice threefold. The study of the safety of the substance NIOCH-14 in the experiments on mice showed that its intragastric administration at a single dose of 150 µg/g had no significant effect on the animals’ appearance and behavior. Moderate changes in hematological parameters were observed (leukopenia and thrombocytosis).

Our findings show good agreement with the data reported by Chen Y. et al. [15], who showed that an oral (p/o) administration of ST-246 to BALB/c mice at doses up to 2 g/kg induced no disorders or damage in the animals. Meanwhile, the optimal effectiveness against the ectromelia virus in BALB/c mice was observed from a p/o administration of ST-246 at a dose of 100 µg/g [31].

The evaluation of safety of the substance NIOCH-14 in our experiments using another type of laboratory animals, Wistar rats, showed that the intragastric administration of a NIOCH-14 suspension at a single 150 µg/g dose to male and female rats had no significant effect on the animals’ appearance and behavior and caused no changes in their hematological parameters. No pathological effects were observed in NZW rabbits after a p/o administration of ST-246 at doses up to 100 µg/g [15].

The subchronic damaging effect of the substance NIOCH-14 was studied in the Wistar rats. A daily intragastric administration of the drug for 30 days to male and female Wistar rats at doses equal to the effective dose (the therapeutic dose, 50 µg/g body weight) and threefold higher than that did not cause animal death. NIOCH-14 did not induce any profound or long-term changes in physiological and hematological parameters, weight, and the macroscopic appearance of the internal organs. A microscopic examination of the internal organs revealed insignificant changes in the histological pattern of the stomach, liver, and kidneys. Our data agree with the findings reported in ref. [34], which showed that a daily p/o administration of 300 µg/g ST-246 to cynomolgus monkeys over 3 months did not cause any pathological processes in the animals. This dose was 100 times higher than the effective dose (3 µg/g) against the monkeypox virus in these animals. Therefore, our findings allow us to infer that the chemically synthesized substance NIOCH-14 is almost safe in experiments on laboratory animals.

As a part of the study to evaluate the pharmacokinetics of the substance NIOCH-14, we have shown that after a p/o administration of a single 50 µg/g dose of NIOCH-14 suspension to mice weighing 12–14 g, the C_max_ of the active metabolite ST-246 in serum reached 2.06 µg/mL at T_max_ = 6.0 h, while T_1/2_ was 4.2 h; in the lungs, the C_max_ of ST-246 reached 2.01 µg/mL at T_max_ = 9.0 h, while T_1/2_ was 12.5 h; in the liver, the C_max_ of ST-246 reached 1.04 µg/mL at T_max_ = 6.0 h, while T_1/2_ was 5.6 h; in the spleen, the C_max_ of ST-246 reached 0.29 µg/mL at T_max_ = 9.0 h, while T_1/2_ = 8.5 h; in the brain, the C_max_ of ST-246 reached 0.25 µg/mL at T_max_ = 9.0 h, while T_1/2_ was 13.2 h; in the kidneys, the C_max_ of ST-246 reached 1.07 µg/mL at T_max_ = 6 h, while T_1/2_ was 5.5 h. The tissue availability (f_T_) of the substance NIOCH-14 upon a p/o administration of a single 50 µg/g dose was 100% for the lungs, 69.6% for the liver, 20.3% for the spleen, 26.8% for the brain, and 63.3% for the kidneys. Our data suggest that, following oral administration, NIOCH-14 (as its active metabolite ST-246) reaches organs that are targets for orthopoxviruses [17,18].

When studying the pharmacokinetics of the chemical compound ST-246, it was previously shown that upon an oral administration of 30 µg/g ST-246 to mice, its plasma C_max_ was 32.5 µg/mL at T_max_ = 2.7 h; T_1/2_ was 2.5 h [28]. As the administered oral dose was increased to 100 µg/g body weight, the plasma C_max_ was 62.2 µg/mL after T_max_ = 1.0 h; T_1/2_ was 2.5 h [28]. It was reported in another study that after an oral administration of 30 µg/g ST-246 to mice, the plasma C_max_ was 38.0 µg/mL and T_1/2_ was 2.4 h, whereas in mice receiving 100 µg/g ST-246 p/o, the plasma C_max_ was as high as 44.0 µg/mL, while T_1/2_ was 2.2 h [3]. It is impossible to properly compare the C_max_, T_max_ and T_1/2_ values obtained in ref. [3,28] to the respective values obtained in our studies, since no methodological details of LC-MS/MS mass spectrometry analysis had been reported in these papers, while in our study, LC-MS/MS was performed in the SRM (selected reaction monitoring) mode. Furthermore, we have determined concentrations of the active metabolite of NIOCH-14 (ST-246) in mouse organ tissues and its tissue availability in compliance with the guidelines for studying the pharmacokinetics of drugs [19], whereas no such studies were performed for the chemical compound ST-246.

Moreover, in another experiment where mice received a single p/o dose of NIOCH-14 (50 µg/g) and a single i/v dose (2 µg/g), the main pharmacokinetic parameters of its active metabolite ST-246 (C_max_, T_max_, T_1/2_, AUC_0-t_, and AUC_0-inf_) had been determined, and F_abs_ was calculated. For the single-dose i/v administration of NIOCH-14 to mice at a dose of 2 µg/g body weight, the serum C_max_ of ST-246 reached 9.52 µg/mL at T_max_ = 0.25 h and T_1/2_ = 2.3 h. After a single-dose p/o administration of the substance NIOCH-14 at a dose of 50 µg/g body weight to mice weighing 18–20 g, the serum C_max_ of ST-246 reached 15.44 µg/mL at T_max_ = 6.0 h and T_1/2_ was 5.7 h. The F_abs_ of the substance of NIOCH-14 was 22.8%.

Since the F_abs_ of the drug is dose-dependent upon oral and intravenous administration, the F_abs_ of NIOCH-14 did not differ from the F_abs_ of the reference drug ST-246. It is known from the research literature that F_abs_ for ST-246 in different mouse strains have ranged from 3.8 to 71.6% [11,15]. Since the F_abs_ values of the substance NIOCH-14 and the reference drug ST-246 determined in our experiments lie in the range of the F_abs_ values for ST-246 reported in refs. [11,15], it is evident that the F_abs_ of NIOCH-14 is not lower than that of ST-246.

The pharmacokinetic parameters of ST-246 according to its concentration in serum (but not organs) have also been determined and reported in earlier studies [11,15,35,36]. A comparison of the blood AUC values after an i/v administration of a single 2 µg/g dose of ST-246 and a p/o administration of a single 10 µg/g dose to mice showed that the F_abs_ of ST-246 was ~31% [11]. When ST-246 was administered in an i/v at a dose of 3 µg/g or p/o at a dose increased to 100 µg/g, the F_abs_ decreased to 20% [15]. Meanwhile, the administration of higher doses of ST-246 (namely, 75 µg/g for i/v administration and 1000 µg/g for p/o administration) substantially reduced F_abs_ to 5.1% [15].

In our studies, when NIOCH-14 was administered in an i/v, the blood C_max_ of its active metabolite ST-246 was expectedly reached almost immediately after the end of the drug administration (T_max_ = 0.25 h). However, the blood T_max_ of ST-246 for the p/o administration of NIOCH-14 at a dose of 50 µg/g was observed later (after 6 h) probably due to the longer duration of drug adsorption in the mouse intestine. The half-life (T_1/2_) of the active metabolite ST-246 in blood upon a p/o administration of NIOCH-14 at a dose of 50 µg/g of body weight was 5.6 h. Our results are consistent with the data reported in ref. [15]. Chen Y. et al. [15] have shown that the C_max_ and T_max_ for ST-246 in serum of BALB/c mice were observed 4.5 h after a single-dose p/o administration of 1000 µg/g ST-246, whereas upon an i/v administration at a dose of 75 µg/g, C_max_ and T_max_ were reached immediately after the 10 min infusion. Furthermore, it has been demonstrated that the half-life (T_1/2_) of ST-246 administered in an i/v and p/o at doses mentioned above were 2.8 and 4.5 h, respectively [15].

## 5. Conclusions

The safety and bioavailability of the substance of the candidate anti-smallpox drug NIOCH-14 was assessed in this study. In terms of its effect on the body, the substance NIOCH-14 can be classified as a low-hazard compound, since a single-dose intragastric administration of NIOCH-14 at a dose of 5 g/kg did not cause death in laboratory mice, intoxication signs, or weight loss in the animals. The single-dose and multiple-dose intragastric administration of the substance NIOCH-14 to mice and rats did not lead to animal death or any critical changes in the hematological parameters nor the microscopic appearance of the animals’ internal organs. The evaluation of the pharmacokinetic parameters of the substance NIOCH-14 according to the concentration of its active metabolite ST-246 in the body of mice revealed that NIOCH-14 administered orally is characterized by tissue availability for the lungs, spleen, kidneys, liver and brain, as well as absolute bioavailability.

Therefore, along with the earlier demonstrated effectiveness against orthopoxviruses (including the variola virus), the substance NIOCH-14 exhibited safety and bioavailability in the experiments on the laboratory animals.

## Figures and Tables

**Table 1:** Hematological parameters 14 days after single-dose intragastric administration of NIOCH-14 to outbred ICR mice (150 µg/g body weight).

Groups of Mice, Drug, Dose	Hemoglobin, g/L	RBC Count, 10^12^/L	Hematocrit, %	Platelet Count, 10^9^/L	ESR, mm/h	WBC Count, 10^9^/L	WBC Differential, %				
							Eosinophils	Neutrophils		Monocytes	Lymphocytes
								Band	Segmented		
Males											
Control 1	154.6 ± 8.5	8.3 ± 0.3	44.3 ± 2.6	1117 ± 68	1.2 ± 0.2	6.4 ± 0.4	3.6 ± 1.6	1.6 ± 0.9	46.8 ± 4.8	2.6 ± 0.7	45.4 ± 5.1
Control 2	153.2 ± 3.1	8.1 ± 0.1	45.3 ± 1.2	1029 ± 63	1.0 ± 0.0	5.9 ± 0.4	3.4 ± 0.9	1.0 ± 0.3	43.4 ± 4.2	4.4 ± 1.0	47.8 ± 3.8
NIOCH-14, 150 µg/g	152.0 ± 5.8	8.2 ± 0.2	44.5 ± 1.8	1101 ± 69	1.2 ± 0.2	4.9 ± 0.3 *	2.6 ± 1.2	1.0 ± 0.4	44.6 ± 7.1	3.0 ± 0.6	48.8 ± 7.7
Females											
Control 1	163.0 ± 2.4	8.9 ± 0.2	46.1 ± 1.0	751 ± 28	1.2 ± 0.2	6.1 ± 0.5	4.4 ± 1.1	0.6 ± 0.2	30.6 ± 6.0	2.8 ± 0.7	61.6 ± 6.5
Control 2	156.6 ± 5.6	8.3 ± 0.3	44.5 ± 1.8	909 ± 53	1.4 ± 0.2	7.1 ± 0.6	2.6 ± 0.9	1.4 ± 0.2	28.0 ± 1.8	3.6 ± 0.9	51.4 ± 2.5
NIOCH-14, 150 µg/g	162.2 ± 3.4	8.7 ± 0.3	40.2 ± 6.1	964 ± 35 *	1.4 ± 0.2	7.1 ± 1.0	3.4 ± 1.1	0.6 ± 0.4	24.0 ± 1.8	5.0 ± 0.6	68.2 ± 3.0

Note: Control 1—the group of mice that received normal saline; Control 2—the group of mice that received the solution for preparing NIOCH-14 suspension containing methylcellulose (0.75%) and Tween 80 (1%). ESR—the erythrocyte sedimentation rate. RBC—red blood cells. WBC—white blood cells. The data are presented as the mean ± mean error (M ± m); each group consisted of five animals. * the differences are statistically significant compared to the parameters in mice that received normal saline, *p* ≤ 0.05.

**Table 2:** Hematological parameters 14 days after single-dose intragastric administration of NIOCH-14 to Wistar rats (150 µg/g body weight).

Groups of Rats, Drug, Dose	Hemoglobin, g/L	RBC Count, 10^12^/L	Hematocrit, %	Platelet Count, 10^9^/L	ESR, mm/h	WBC Count, 10^9^/L	WBC Differential, %				
							Eosinophils	Neutrophils		Monocytes	Lymphocytes
								Band	Segmented		
Males											
Control 1	140.0 ± 2.2	6.8 ± 0.1	38.2 ± 0.8	678 ± 32	1.0 ± 0	10.7 ± 1.2	2.0 ± 0.3	0.8 ± 0.2	33.6 ± 6.9	5.2 ± 1.3	58.4 ± 6.4
Control 2	137.2 ± 3.0	6.5 ± 0.1	37.1 ± 0.7	743 ± 73	3.8 ± 1.6	6.96 ± 0.5	3.8 ± 0.7	1.4 ± 0.2	37.6 ± 6.3	4.4 ± 0.7	52.8 ± 6.3
NIOCH-14, 150 µg/g	129.0 ± 6.4	6.47 ± 0.3	34.7 ± 1.6	668 ± 69	3.4 ± 1.2	10.4 ± 1.9	1.8 ± 1.4	1.6 ± 0.9	40.6 ± 3.1	6.8 ± 1.2	49.2 ± 3.3
Females											
Control 1	143.2 ± 2.4	6.6 ± 0.2	37.5 ± 0.7	767 ± 55	2.8 ± 1.4	9.76 ± 0.5	1.6 ± 0.8	1.0 ± 0.3	25.6 ± 5.7	4.2 ± 0.2	67.6 ± 5.4
Control 2	137.8 ± 3.5	6.5 ± 0.2	35.9 ± 0.6	802 ± 31	5.2 ± 1.8	10.0 ± 0.6	3.2 ± 0.7	1.2 ± 0.7	37.6 ± 7.4	4.4 ± 0.9	53.6 ± 8.1
NIOCH-14, 150 µg/g	137.6 ± 4.0	6.4 ± 0.2	35.7 ± 0.9	756 ± 47	2.2 ± 0.4	8.68 ± 0.7	2.8 ± 0.7	1.2 ± 0.5	29.4 ± 2.0	6.4 ± 1.1	60.2 ± 2.5

Note: Control 1—the group of rats that received normal saline; Control 2—the group of rats that received the solution for preparing NIOCH-14 suspension containing methylcellulose (0.75%) and Tween 80 (1%). ESR—the erythrocyte sedimentation rate. RBC—red blood cells. WBC—white blood cells. The data are presented as the mean ± mean error (M ± m); each group consisted of five animals.

**Table 3:** Hematological parameters 1 day after the end of intragastric administration of NIOCH-14 to Wistar rats once daily during 30 days.

Groups of Rats, Drug, Dose	Hemoglobin, g/L	RBC Count, 10^12^/L	Hematocrit, %	Platelet Count, 10^9^/L	ESR, mm/h	WBC Count, 10^9^/L	WBC Differential, %				
							Eosinophils	Neutrophils		Monocytes	Lymphocytes
								Band	Segmented		
Males											
Control 1	144.8 ± 4.9	7.0 ± 0.2	38.2 ± 1.3	725 ± 36	2.6 ± 0.2	10.6 ± 0.9	1.2 ± 0.5	0.8 ± 0.4	39.0 ± 3.4	4.6 ± 0.8	54.4 ± 3.7
Control 2	158.6 ± 2.1	7.6 ± 0.2	42.3 ± 0.5	828 ± 51	2.4 ± 0.2	6.8 ± 0.5	4.2 ± 1.0	2.2 ± 1.1	44.4 ± 3.1	6.0 ± 1.0	43.2 ± 4.1
NIOCH-14, 50 µg/g	144.3 ± 3.2	7.0 ± 0.1	38.2 ± 0.9	795 ± 24	1.8 ± 0.3	7.4 ± 0.3	3.2 ± 1.4	1.3 ± 0.4	41.8 ± 2.8	5.5 ± 0.8	48.2 ± 4.0
NIOCH-14, 150 µg/g	146.0 ± 2.6	6.9 ± 0.2	38.1 ± 0.8	723 ± 34	1.8 ± 0.3	9.1 ± 0.8	2.3 ± 0.6	1.0 ± 0.6	38.2 ± 5.3	3.8 ± 0.8	54.7 ± 5.6
Females											
Control 1	144.4 ± 2.0	6.7 ± 0.2	37.0 ± 0.7	793 ± 54	1.6 ± 0.2	6.5 ± 0.7	3.8 ± 1.2	0.6 ± 0.4	52.8 ±4.7	4.2 ± 0.4	38.6 ± 5.2
Control 2	141.4 ± 1.3	6.7 ± 0.1	37.6 ± 0.4	712 ± 40	1.6 ± 0.4	7.2 ± 0.8	4.8 ± 0.9	1.0 ± 0.6	40.8 ± 3.5	4.2 ± 0.4	49.2 ± 3.8
NIOCH-14, 50 µg/g	146.3 ± 1.0	6.9 ± 0.1	38.7 ± 0.3	701 ± 47	1.3 ± 0.2	6.6 ± 0.5	3.5 ± 1.1	0.5 ± 0.3	41.3 ± 4.0	4.7 ± 1.0	49.8 ± 3.0
NIOCH-14, 150 µg/g	141.5 ± 2.9	6.7 ± 0.1	37.4 ± 0.8	742 ± 39	1.7 ± 0.3	6.1 ± 0.6	1.8 ± 0.5	0.8 ± 0.3	45.2 ± 1.7	6.2 ± 0.5	46.0 ± 1.6

Note: Control 1—the group of rats that received normal saline; Control 2—the group of rats that received the solution for preparing NIOCH-14 suspension containing methylcellulose (0.75%) and Tween 80 (1%). ESR—the erythrocyte sedimentation rate. RBC—red blood cells. WBC—white blood cells. The data are presented as the mean ± mean error (M ± m); each group consisted of five animals.

**Table 4:** Pharmacokinetic parameters of the active metabolite ST-246 in mouse serum and organs upon single-dose oral administration of NIOCH-14 (50 µg/g).

Parameters (Measurement Units)	Blood Serum	Lungs	Liver	Spleen	Brain	Kidneys
T_1/2_ (h)	4.2	12.5	5.6	8.5	13.2	5.5
T_max_ (h)	6	9	6	9	9	6
C_max_ (ng/mL)	2058 ± 641	2009 ± 1746	1043 ± 514	286 ± 158	252 ± 38	1072 ± 453
AUC_0-t_ (ng/mL) × h	22,666	21,880	15,026	3989	4277	13,813
AUC_0-inf_ (ng/mL) × h	23,551	23,972	16,391	4786	6314	14,910
f_T_ (%)	-	100	69.6	20.3	26.8	63.3

Note: C_max_ is presented as M ± SD; each group consisted of six animals.

**Table 5:** Pharmacokinetic parameters (in the serum) of the substance NIOCH-14 and the reference drug ST-246 administered as a single dose (2 µg/g body weight) intravenously (i/v) and oral (p/o) to mice at a dose of 50 µg/g body weight.

Parameters (Units of Measurement)	Administration of NIOCH-14		Administration of ST-246	
	i/v, 2 µg/g	p/o, 50 µg/g	i/v, 2 µg/g	p/o, 50 µg/g
T_1/2_ (h)	2.3	5.7	2.0	3.4
T_max_ (h)	0.25	6	0.25	3
C_max_ (ng/mL)	9515 ± 3903	15,439 ± 3373	13,200 ± 4287	15,495 ± 3227
AUC_0-t_ (ng/mL) × h	24,918	141,883	34,254	103,661
AUC_0-inf_ (ng/mL) × h	25,038	142,220	34,318	105,405
F_abs_ (%)	-	22.8	-	12.1

Note: C_max_ is presented as M ± SD; each group consisted of six animals.

## Data Availability

Not applicable.

## Abbreviation

Absolute Bioavailability of the Drug = F_abs_
Area under the Curve “Concentration-Time” = AUC
Area under the Curve “Concentration-Time” from 0 to time t = AUC_0-t_
Area under the Curve “Concentration-Time” from 0 to ∞ = AUC_0-inf_
Cowpox Virus = CPXV
Dimethyl Sulfoxide = DMSO
Dose of the Drug at Intravenous (i/v) Administration = D_i/v_
Dose of the Drug at Per os (p/o) Administration = D_p/o_
Ectromelia Virus = ECTV
Error of the Mean = m
Erythrocyte Sedimentation Rate = ESR
Extracellular Enveloped Virus = EEV
Food and Drug Administration = FDA
Half-life of the Drug = T_1/2_
Intracellular Mature Virus = IMV
Intravenous, intravenously = i/v
50% Lethal Dose = LD_50_
Maximum Drug Concentration = C_max_
Mean Value = M
Monkeypox Virus = MPXV
Multiple Reaction Monitoring = MRM
Oral, Per os = p/o
Rabbitpox Virus = RPXV
Red Blood Cells = RBC
Selected Reaction Monitoring = SRM
Standard Deviation = SD
State Standard = GOST
Tandem Mass Spectrometry—MS/MS and Liquid Chromatography—LC = LC-MS/MS
Time to Reach Maximum Drug Concentration = T_max_
Tissue Availability of the Drug for the Organs = f_T_
Vaccinia Virus = VACV
Variola Virus = VARV
White Blood Cells = WBC
